# Genetic Determinants of On-Aspirin Platelet Reactivity: Focus on the Influence of *PEAR1*

**DOI:** 10.1371/journal.pone.0111816

**Published:** 2014-10-31

**Authors:** Morten Würtz, Peter H. Nissen, Erik Lerkevang Grove, Steen Dalby Kristensen, Anne-Mette Hvas

**Affiliations:** 1 Department of Cardiology, Aarhus University Hospital, Aarhus, Denmark; 2 Department of Internal Medicine, Regional Hospital West Jutland, Herning, Denmark; 3 Department of Clinical Biochemistry, Aarhus University Hospital, Aarhus, Denmark; University Hospital Medical Centre, Germany

## Abstract

**Background:**

Platelet aggregation during aspirin treatment displays considerable inter-individual variability. A genetic etiology likely exists, but it remains unclear to what extent genetic polymorphisms determine platelet aggregation in aspirin-treated individuals.

**Aim:**

To identify platelet-related single nucleotide polymorphisms (SNPs) influencing platelet aggregation during aspirin treatment. Furthermore, we explored to what extent changes in cyclooxygenase-1 activity and platelet activation may explain such influence.

**Methods:**

We included 985 Danish patients with stable coronary artery disease treated with aspirin 75 mg/day mono antiplatelet therapy. Patients were genotyped for 16 common SNPs in platelet-related genes using standard PCR-based methods (TaqMan). Platelet aggregation was evaluated by whole blood platelet aggregometry employing Multiplate Analyzer (agonists: arachidonic acid and collagen) and VerifyNow Aspirin. Serum thromboxane B_2_ was measured to confirm aspirin adherence and was used as a marker of cyclooxygenase-1 activity. Soluble P-selectin was used as marker of platelet activation. Platelet aggregation, cyclooxygenase-1 activity, and platelet activation were compared across genotypes in adjusted analyses.

**Results:**

The A-allele of the rs12041331 SNP in the platelet endothelial aggregation receptor-1 (*PEAR1*) gene was associated with reduced platelet aggregation and increased platelet activation, but not with cyclooxygenase-1 activity. Platelet aggregation was unaffected by the other SNPs analyzed.

**Conclusion:**

A common genetic variant in *PEAR1* (rs12041331) reproducibly influenced platelet aggregation in aspirin-treated patients with coronary artery disease. The exact biological mechanism remains elusive, but the effect of this polymorphism may be related to changes in platelet activation. Furthermore, 14 SNPs previously suggested to influence aspirin efficacy were not associated with on-aspirin platelet aggregation.

**Clinical Trial Registration:**

ClinicalTrials.gov NCT01383304

## Introduction

Low-dose aspirin substantially reduces the risk of recurrent arterial thrombosis [1], yet one fifth of aspirin-treated patients suffer recurrent cardiovascular events. This may reflect that some patients do not derive adequate platelet inhibition from aspirin [1]–[2]. The reasons for reduced effect of aspirin include clinical, biological, pharmacodynamic, and genetic elements [3].

Epidemiological and family studies have repeatedly shown that genetic predisposition accounts for 40% to 60% of the risk for coronary artery disease (CAD) [4]. Moreover, heritable factors account for an estimated 30% of the variation in innate platelet reactivity [5], and genetic variability is an important contributor to residual platelet reactivity during aspirin treatment [6]. Therefore, a biological basis for familial clustering of aspirin response phenotypes may exist, but delineating the specific genetic architecture that predisposes to reduced effect of aspirin remains challenging.

Aspirin irreversibly inhibits cyclooxygenase-1 (COX-1) thereby reducing the conversion of arachidonic acid to thromboxane (TX) A_2_. It follows that the functions of aspirin are elicited mainly through the thromboxane receptor [7]. However, given the considerable interdependency of platelet activation pathways, and the fact that aspirin has effects independent of COX-1 [8]–[9], the effect of aspirin may also be susceptible to genetically determined changes of various other receptors. The platelet surface hosts a panel of receptors mediating platelet activation, and ultimately they all converge towards the glycoprotein (GP) IIb/IIIa fibrinogen receptor complex. In the search for genetic mechanisms to explain inadequate platelet inhibition by aspirin, especially the IIIa subunit of this complex has been scrutinized [10]. However, genetic variability in a range of other receptors and key enzymes may also contribute. Most recently, a common intronic G→A single nucleotide polymorphism (SNP) in the *PEAR1* locus on chromosome 1 has been linked with platelet expression of the platelet endothelial aggregation receptor 1 (PEAR1) [11] and with platelet aggregation [11]–[13]. PEAR1 was described for the first time by Nanda *et al*., who identified this receptor as a transmembrane molecule participating in contact-induced platelet activation resulting from interaction between platelets [14]. Phosphorylation of PEAR1 seems to reinforce activation of the GP IIb/IIIa fibrinogen receptor thereby promoting platelet aggregation [15], but the exact function of this receptor remains largely unknown and its surface ligand has not yet been identified.

The main objective of this study was to identify platelet-related genetic variants influencing platelet aggregation in aspirin-treated patients with CAD. Furthermore, we explored biological mechanisms that may explain such influence.

## Methods

### Ethics Statement

The study was conducted in accordance with the Helsinki II declaration, and the study protocol was approved by the Central Denmark Region Committees on Biomedical Research Ethics (M-2009-0110). Written informed consent was obtained from all participants, none of whom were remunerated for their study participation. The study is registered at www.clinicaltrials.gov (NCT01383304).

### Study Population

We included a total of 985 Danish patients with CAD. From November 2007 through January 2011 patients were recruited from the Western Denmark Heart Registry, which collects data on all interventional procedures performed in interventional centers in the western part of Denmark [16]. Diagnoses of CAD were based on angiographically documented coronary artery stenosis. Information on co-medication was obtained on the day of blood sampling.

Exclusion criteria were aspirin intolerance, any acute disease, use of anticoagulants or any drugs other than aspirin known to affect platelet function (*i.e.* thienopyridines, ticagrelor, dipyridamol, and non-steroidal anti-inflammatory drugs), platelet count <120×10^9^/L, pregnancy, any ischemic event or revascularization procedure (percutaneous coronary intervention or coronary artery bypass grafting) within the previous 12 months, and inability to give informed consent.

### Study Medication and Compliance

All patients were on permanent aspirin therapy upon study enrollment. In order to ensure compliance and avoid pharmacokinetic heterogeneity, all patients received a tablet box containing a one-week supply of the study medication, *i.e.* one 75 mg non-enteric coated aspirin tablet (Hjerdyl; Sandoz, Copenhagen, Denmark) for each of the last seven days prior to blood sampling. The seventh tablet was ingested exactly one hour before blood sampling. Adherence to aspirin was confirmed by measurement of serum TXB_2_.

### Blood Sampling

Standardized blood sampling was performed between 8 AM and noon with patients resting for 30 minutes before sampling. Samples were drawn from an antecubital vein into evacuated tubes through a 19-gauge butterfly needle using a minimum of stasis. The first tube was discarded.

### Platelet Aggregometry

The primary outcome variable was platelet aggregation evaluated one hour after aspirin intake. Platelet aggregometry was performed using two different whole blood tests; multiple electrode aggregometry (Multiplate Analyzer; Roche Diagnostics International LDT, Rotkreuz, Switzerland) and VerifyNow Aspirin (Accumetrics Inc., San Diego, CA, USA). Blood was collected in 3.6 mL (Multiplate Analyzer) and 2.7 mL (VerifyNow Aspirin) tubes containing 3.2% sodium citrate (Terumo, Leuven, Belgium), and all analyses were completed within two hours of sampling.

Multiplate Analyzer is an impedance aggregometer used for multiple electrode aggregometry [17]. Agonist solutions were delivered using an automatic pipette and contained arachidonic acid or collagen at final agonist concentrations of 1.0 mmol/L and 3.2 µg/mL (Roche Diagnostics International LDT, Rotkreuz, Switzerland). Aggregation was reported as arbitrary aggregation units plotted against time and the area under the aggregation curve was measured (aggregation units × min). The VerifyNow instrument is based on turbidimetric optical detection of platelet aggregation, and the VerifyNow Aspirin assay employs arachidonic acid as the agonist. Results are reported as arbitrary Aspirin Reaction Units.

### Candidate Genes

The primary exposure variables were numbers of risk alleles according to the SNPs being investigated. The selection of candidate genes and SNPs was based on a thorough review of existing literature, as previously described [18]. We identified a total of 18 SNPs with suggested influence on aspirin-dependent platelet and/or patient phenotype. Attempts to genotype rs1126643 and rs2243093 were unsuccessful and rs2046934 did not meet the Hardy-Weinberg criteria. Accordingly, these three SNPs were excluded from further statistical analyses leaving a total of 15 SNPs for final data interpretation. All analyses were performed by trained laboratory technicians blinded to the outcome results.

### Genotyping

Genomic DNA was isolated from EDTA stabilized peripheral blood using the Maxwell 16 Blood DNA Purification Kit (Promega Corporation, WI, USA) according to the manufacturer's instructions. After testing the performance of each assay on a limited number of samples, genotyping of the entire cohort was performed using TaqMan SNP genotyping assays (Life Technologies Corporation, NY, USA). Thermocycling was performed in a GeneAmp PCR System 9700 (Life Technologies Corporation). The thermal cycling profile comprised an initial denaturation at 95°C for 10 min, followed by 40–50 cycles (depending on signal strength) at 95°C for 15 sec and at 60°C for one min. Four negative controls were included in each 96-well plate. Collection of fluorescent signals was performed using the plate read function in an Mx3005P Real-Time PCR system (Agilent Technologies Inc., CA, USA) and final discrimination of genotypes was verified by manual inspection of scatterplots.

### Serum Thromboxane B_2_ and P-Selectin

TXB_2_ produced *ex vivo* during whole blood clotting is an index of platelet COX-1 activity [7]. Serum TXB_2_ thus reflects platelet inhibition exerted by aspirin and is a highly specific marker of aspirin adherence. P-selectin is a platelet surface adhesion molecule and was used to evaluate platelet activation. Commercially available enzyme-linked immunosorbent assays were used according to the manufacturers' instructions to determine levels of serum TXB_2_ (Cayman Chemical, Ann Arbor, MI, USA) and soluble serum P-selectin (R&D Systems Europe, Abingdon, UK).

### Statistics

Summary statistics and frequencies were generated using the Stata 12.1 software package (Stata Corp LP, TX, USA), and graphics were performed in GraphPad Prism version 6.03 (GraphPad Software, La Jolla, CA, USA). Continuous data are presented as mean (variance) or median (interquartile range). Distributions of discrete variables were compared with the χ^2^ test and are presented as counts and percentages. The goodness-of-fit χ^2^ test was used to compare observed frequencies of alleles and genotypes against the Hardy-Weinberg equilibrium prediction. Genotype data were considered according to the general genetic model, in which three distinct genotypes are retained [19]. It follows that all tests evaluating platelet aggregation as a function of genotype were conducted assuming an additive genetic model. Trends in platelet aggregation across genotypes were tested using multivariable linear regression adjusting for the following baseline variables and cardiovascular risk factors: Age, sex, smoking, body mass index, previous myocardial infarction, diabetes, proton pump inhibitor use, and platelet count. The selection of variables included in the regression model was based on an evaluation of plausible predictors of the outcome (*i.e.* platelet aggregation). Acknowledging the relatively low number of patients homozygous for the variant alleles, these subjects were pooled with heterozygotes for regression analysis. Platelet aggregation was analyzed retaining the original scaling of continuous variables to preserve statistical power and to minimize the risk of type II errors. Where appropriate, calculations were made using logarithm-transformed data. Listwise deletion was used to account for missing data. To account for multiple testing, associations between aggregation levels and genotypes were considered significant at the Bonferroni-adjusted level of p<0.0011 (0.05/[15 SNPs × 3 aggregation phenotypes]).

## Results

Baseline characteristics, concomitant drug use, and cardiovascular risk factors of the 985 enrolled patients are presented in Table 1. Patients were 65±9 years old and predominantly men (78%). Most patients had a history of previous percutaneous coronary intervention (94%) and myocardial infarction (81%), and one fourth were diabetics. TXB_2_ levels were suppressed in all patients (geometric mean TXB_2_ 0.98 [95% confidence interval 0.93 to 1.04] ng/mL, range 0.02 to 26.4 ng/mL) confirming near maximal aspirin-induced suppression of platelet COX-1 activity. Adherence to aspirin thus was 100%.

**Table 1:** Characteristics of the study population.

**Demographics**	
Age, years	65±9
Male sex, n (%)	771 (78)
Danish citizen, n (%)	985 (100)
**Risk factors**	
Smoking, n (%)^1^	
Habitual smoker	212 (22)
Ex-smoker	494 (50)
Non-smoker	279 (28)
Systolic blood pressure, mmHg	142±20
Diastolic blood pressure, mmHg	83±12
Family history of ischemic heart disease, n (%)^2^	384 (39)
Body mass index, kg/m^2^	27.7±4.4
**Biochemistry**	
P-Creatinine, µmol/L^3^	84.2 (82.8–85.6)
B-Platelet count, 10^9^/L^3^	226 (222–230)
B-Hemoglobin, mmol/L	8.8±0.8
Hematocrit, %	42.6±3.2
**Medical history, n (%)**	
Coronary artery disease	985 (100)
Myocardial infarction	802 (81)
Percutaneous coronary intervention	925 (94)
Coronary artery bypass grafting	135 (14)
Stroke	58 (6)
Diabetes	259 (26)
**Medication, n (%)**	
Aspirin	985 (100)
Statins	895 (91)
β-blockers	738 (75)
Angiotensin-converting enzyme inhibitors	444 (45)
Angiotensin-II receptor antagonists	156 (16)
Calcium antagonists	215 (22)
Diuretics	294 (30)
Proton pump inhibitors	117 (12)

Values are mean ± standard deviation or counts (%) unless otherwise stated. ^1^Self-reported history of smoking cigarette, pipe, or cigar. ^2^First-degree relatives with onset of ischemic heart disease before the age of 60 years. ^3^Logarithm transformed for analysis and back-transformed for presentation as geometric mean (95% confidence interval).

(n = 985).

### Genotype Distribution

Genotype distributions were consistent with the Hardy-Weinberg expectations except for rs2046934 in the *P2RY12* gene (p = 0.008) (Table 2). Accordingly, rs2046934 genotype data were not tested for association with platelet aggregation. In general, genotype distributions and minor allele frequencies were as expected according to the NCBI SNP database (dbSNP) [20] except that the minor allele frequency of rs12041331 was lower than expected, with 8% in our cohort and 28% in dbSNP [20].

**Table 2:** Genotype distributions in 985 aspirin-treated patients with stable coronary artery disease.

Gene	Gene product	Nucleotide change^1^	Ref SNP ID^2^	Minor allele	MAF	Call rate, %	Hardy-Weinberg equilibrium^3^
*ITGB3*	GP IIIa	Pl^A1/A2^ (T1565→C)	rs5918	C	0.17	98.8	0.98
*PTGS1*	COX-1	A-842→G/C50→T	rs10306114	G	0.07	98.8	0.72
*PTGS1*	COX-1	C22→T	rs1236913	T	0.08	98.8	0.34
*TBXA2R*	TXA_2_ receptor	C924→T	rs4523	A	0.33	98.6	1.0
*TBXA2R*	TXA_2_ receptor	C795→T	rs1131882	A	0.14	98.6	0.96
*P2RY1*	P2Y_1_	C893→T	rs1065776	T	0.04	98.6	0.98
*P2RY1*	P2Y_1_	A1622→G	rs701265	G	0.14	98.8	1.0
*P2RY12*	P2Y_12_	H2 haplotype	rs6809699^4^	A	0.15	98.5	0.35
*P2RY12*	P2Y_12_	H2 haplotype	rs10935838^d4^	A	0.15	96.3	0.35
*P2RY12*	P2Y_12_	H2 haplotype	rs2046934^4^	G	0.18	98.5	0.008
*GP1BA*	GP Iba	C145→T (HPA-2)	rs6065	T	0.07	98.6	0.90
*GP6*	GP VI	T13254→C	rs1613662	G	0.17	98.6	0.94
*PLA2G7*	Platelet-activating factor acetylhydrolase	G994→T	rs1051931	A	0.21	96.8	0.84
*F13A1*	Coagulation factor XIII	G34→T	rs5985	A	0.26	98.6	0.78
*PEAR1*	Platelet endothelial aggregation receptor-1	A→G	rs12041331	A	0.08	97.1	0.98
*PEAR1*	Platelet endothelial aggregation receptor-1	A→C	rs2768759	A	0.26	98.6	0.38

1 Nucleotide change or alternative name as most often termed in the existing literature. ^2^According to the NCBI SNP database, dbSNP: http://www.ncbi.nlm.nih.gov/projects/SNP/ (accessed on August 15, 2014). ^3^P-values refer to the goodness-of-fit χ^2^ test for deviation from the Hardy-Weinberg equilibrium. ^4^Included in the H1/H2 haplotype constituted by rs10935838, rs2046934, rs5853517, and rs6809699 [35]. COX: cyclooxygenase, GP: glycoprotein, MAF: minor allele frequency, SNP: single nucleotide polymorphism, TXA_2_: thromboxane A_2_.

### Platelet Aggregation According to Genotype

**Figure 1:**
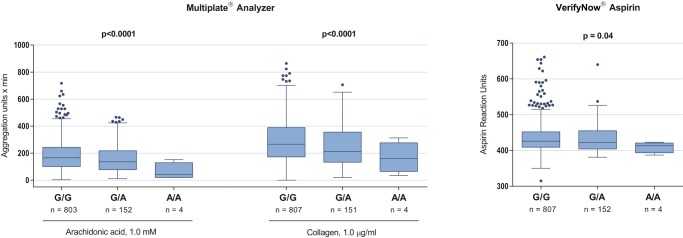
Association of *PEAR1* rs12041331 genotype with on-aspirin platelet aggregation assessed by multiple electrode aggregometry (Multiplate Analyzer) and VerifyNow Aspirin. Comparisons of platelet aggregation levels across genotypes were adjusted for the following baseline variables and cardiovascular risk factors: Age, sex, smoking, body mass index, previous myocardial infarction, diabetes, proton pump inhibitor use, and platelet count. Patients homozygous for the A allele were pooled with heterozygotes for regression analysis. Horizontal lines and boxes indicate median with interquartile range. Whiskers indicate ±1.5 interquartile range and points beyond whiskers indicate outliers.

In Table 3, platelet aggregation levels are presented according to genotype for each SNP. Associations between aggregation levels and genotypes were assessed employing the Bonferroni-adjusted significance level of p<0.0011. Patients homozygous AA or heterozygous GA for the rs12041331 SNP displayed decreased on-aspirin platelet aggregation in response to arachidonic acid and collagen (Figure 1). The major difference was between the AA and GG genotypes and results were robust across platelet function tests, although the signal was stronger when using multiple electrode aggregometry compared to the VerifyNow Aspirin assay. None of the other 14 SNPs subjected to statistical analysis revealed any association with on-aspirin platelet aggregation that was consistent across platelet function tests or agonists.

**Table 3:** On-aspirin platelet aggregation according to genotype in 985 patients with stable coronary artery disease.

			Platelet aggregation				P-values		
							(multivariable-adjusted)		
Gene	Polymorphism	Genotype	MEA (AU × min)		MEA (AU × min)	VerifyNow Aspirin	MEA	MEA	VerifyNow
		(%)	AA 1.0 mmol/L		Collagen 1.0 µg/mL	(Aspirin Reaction units)	AA	Collagen	
*ITGB3*	rs5918	TT (68.5)	145 (137–154)	237 (226–249)		433 (430–436)			
		TC (28.5)	147 (135–160)	250 (232–270)		434 (430–438)	0.98	0.43	0.71
		CC (3.0)	158 (127–197)	244 (200–299)		425 (410–439)			
*PTGS1*	rs10306114	AA (87.0)	147 (140–154)	240 (229–250)		433 (430–435)			
		AG (12.5)	140 (120–164)	250 (223–282)		437 (430–444)	0.12	0.55	0.45
		GG (0.5)	218 (−)	249 (−)		465 (−)			
*PTGS1*	rs1236913	CC (86.0)	145 (138–153)	243 (233–254)		433 (431–436)			
		CT (13.0)	150 (132–169)	232 (208–259)		432 (426–439)	0.43	0.14	0.68
		TT (1.0)	144 (94–220)	190 (115–312)		417 (404–431)			
*TBXA2R*	rs4523	GG (44.5)	147 (137–158)	238 (224–254)		432 (428–435)			
		GA (44.5)	142 (133–152)	238 (224–253)		434 (431–438)	0.78	0.71	0.82
		AA (11.0)	160 (137–185)	266 (236–301)		434 (424–444)			
*TBXA2R*	rs1131882	GG (74.5)	145 (137–154)	238 (227–250)		431 (429–434)			
		GA (23.5)	146 (132–160)	244 (224–266)		439 (433–445)	0.83	0.86	0.65
		AA (2.0)	208 (166–260)	335 (249–451)		430 (402–460)			
*P2RY1*	rs1065776	CC (92.5)	147 (140–154)	241 (231–252)		433 (431–436)			
		CT (7.0)	137 (112–167)	242 (207–282)		435 (427–443)	0.68	0.93	0.87
		TT (0.5)	133 (−)	181 (−)		402 (−)			
*P2RY1*	rs701265	AA (75.0)	146 (138–154)	242 (231–254)		433 (430–436)			
		AG (23)	146 (132–160)	238 (219–259)		433 (429–438)	0.17	0.31	0.22
		GG (2)	154 (118–201)	239 (188–304)		446 (429–465)			
*P2RY12*	rs6809699	CC (72.0)	143 (135–151)	236 (225–248)		432 (429–434)			
		CA (26.5)	154 (141–169)	260 (240–281)		437 (432–442)	0.50	0.37	0.18
		AA (1.5)	162 (123–214)	195 (146–262)		433 (419–447)			
*P2RY12*	rs10935838	GG (71.5)	142 (134–151)	235 (224–247)		431 (429–434)			
		GA (27.0)	155 (143–169)	261 (242–281)		438 (433–442)	0.33	0.30	0.11
		AA (1.5)	162 (123–214)	195 (146–262)		433 (419–447)			
*GP1BA*	rs6065	CC (87.5)	147 (140–155)	239 (229–250)		433 (430–435)			
		CT (12.0)	144 (128–161)	257 (231–285)		435 (427–443)	0.24	0.90	0.98
		TT (0.5)	72 (27–192)	207 (123–349)		427 (407–449)			
*GP6*	rs1613662	AA (69.5)	148 (140–157)	238 (226–250)		433 (430–436)			
		AG (27.5)	143 (131–157)	251 (232–271)		433 (428–438)	0.72	0.08	0.62
		GG (3.0)	112 (76–166)	236 (182–306)		431 (418–444)			
*PLA2G7*	rs1051931	GG (62.5)	146 (138–155)	244 (232–257)		432 (429–435)			
		GA (33.5)	145 (133–157)	232 (216–250)		433 (429–438)	0.73	0.10	0.13
		AA (4.0)	154 (123–192)	264 (221–315)		441 (425–457)			
*F13A1*	rs5985	CC (55.5)	146 (137–155)	234 (221–247)		432 (429–435)			
		CA (37.0)	146 (135–157)	253 (237–270)		435 (431–439)	0.72	0.19	0.66
		AA (7.5)	150 (128–177)	237 (207–271)		434 (426–442)			
*PEAR1*	rs12041331	GG (84.0)	150 (143–158)	249 (239–260)		434 (431–436)			
		GA (15.5)	126 (111–143)	203 (181–229)		431 (424–437)	<0.0001	<0.0001	0.04
		AA (0.5)	46 (10–203)	97 (11–859)		407 (363–456)			
*PEAR1*	rs2768759	CC (55.0)	147 (138–157)	246 (232–260)		433 (430–436)			
		CA (37.5)	146 (136–157)	239 (225–254)		434 (430–438)	0.39	0.30	0.84
		AA (7.5)	136 (112–165)	220 (183–265)		431 (422–441)			

Values are geometric mean (95% confidence interval) or counts (%). Platelet aggregation data were logarithm transformed for analysis and back-transformed for presentation. All analyses are adjusted for the following baseline variables and cardiovascular risk factors: Age, sex, smoking, body mass index, previous myocardial infarction, diabetes, proton pump inhibitor use, and platelet count. P-values refer to differences in platelet aggregation across genotypes. Patients homozygous for the variant allele were pooled with heterozygotes for regression analysis. AA: arachidonic acid, AU: aggregation units, MEA: multiple electrode aggregometry (Multiplate Analyzer).

### COX-1 Activity and Platelet Activation According to PEAR1 rs12041331 Genotype

To further explore possible mechanisms explaining the association between rs12041331 genotype and on-aspirin platelet aggregation, we measured markers of COX-1 activity and platelet activation (Table 4). Levels of TXB_2_ (p = 0.28) and P-selectin (p = 0.05) were numerically higher in A allele carriers, but results were not significant.

**Table 4:** Cyclooxygenase-1 activity and platelet activation according to *PEAR1* rs12041331 genotype.

rs12041331 genotype	Thromboxane B_2_ (ng/mL)^1^	P-value	P-selectin (ng/mL)	P-value
GG	0.97 (0.91–1.03)		74.3±24.9	
GA	1.06 (0.90–1.24)	0.28	78.6±28.4	0.05
AA	1.15 (0.15–8.81)		78.3±18.7	

Values are mean ± standard deviation unless otherwise stated. Patients homozygous for the variant allele were pooled with heterozygotes and analyses are made according to the unpaired *t*-test. ^1^Logarithm transformed for analysis and back-transformed for presentation as geometric mean (95% confidence interval).

## Discussion

In a cardiovascular risk population on aspirin mono antiplatelet therapy, we investigated the association between on-aspirin platelet aggregation and 15 SNPs in platelet-related genetic loci. The principal findings of our study were 1) that a common intronic SNP in the *PEAR1* locus (rs12041331) was associated with low on-aspirin platelet aggregation, 2) that this SNP may also influence the level of platelet activation, but not COX-1 activity, and 3) that a total of 14 SNPs previously suggested to influence aspirin efficacy were not associated with on-aspirin platelet aggregation.

Carriers of the *PEAR1* rs12041331 AA or GA genotype displayed reduced platelet aggregation compared to their GG counterparts. The association remained after adjustment for baseline variables and cardiovascular risk factors. Interestingly, the variation in platelet aggregation across rs12041331 genotypes was not accompanied by corresponding variation patterns in terms of TXB_2_ levels. The pronounced effect of rs12041331 on platelet aggregation in response to arachidonic acid and collagen, but not on TXB_2_ levels, may indicate that PEAR1 signals through pathways distinct from those mediated by COX-1. Thereby, our study extends existing data and adds new pathophysiological insight to previous studies [11]–[13], particularly the one by Lewis *et al*. in which rs12041331 A allele carrier status was identified as an independent determinant of on-aspirin platelet aggregation and adverse clinical outcomes [13]. Whether the association between on-aspirin platelet aggregation and rs12041331 observed in our study impacts on the risk of thrombotic events was beyond the scope of our study. Therefore, the paradoxical finding by Lewis *et al*. that the rs12041331 A allele was associated with reduced on-aspirin platelet aggregation and increased cardiovascular event rates merits further investigation.

Reduced effect of aspirin is a complex phenotypic trait, and although aspirin targets the COX-1 specific pathway of platelet aggregation, there are studies indicating that aspirin may exert antiplatelet effects beyond mere COX-1 acetylation [8]–[9]. In addition, concomitant use of P2Y_12_ receptor antagonists like clopidogrel may potentiate COX-1 inhibition in aspirin-treated patients [21]. Therefore, our study was restricted to patients on aspirin mono antiplatelet therapy (*i.e.* no other antithrombotic drugs were allowed) in order to exclude synergistic effects of co-administered antithrombotic drugs as a potential explanation for our findings. Furthermore, as non-adherence is the primary cause of insufficient platelet inhibition in patients prescribed aspirin [22], we verified aspirin adherence by use of TXB_2_, which is considered the most sensitive marker for COX-1 activity [7].

It is conceivable to find an association between a genetic variant and a phenotype when the genetic variant itself has a proven functional effect on the trait of interest, or when the variant is in linkage disequilibrium with another genetic variant having such effect. Our study does not clarify whether the rs12041331 polymorphism itself modifies on-aspirin platelet aggregation, or whether it is in linkage disequilibrium with a yet unidentified causal *PEAR1* variant. According to a recent study, rs12041331 does not seem to be in linkage disequilibrium with distinct variants with particular effect on platelet aggregation, however, a panel of combined synonymous and non-synonymous exonic variants in *PEAR1* may influence platelet aggregation more strongly than rs12041331 [23]. To further explore possible mechanisms explaining the observed association between rs12041331 genotype and on-aspirin platelet aggregation, we evaluated soluble P-selectin levels according to rs12041331 genotype. We found that rs12041331 A allele carriers displayed numerically higher levels of soluble P-selectin, although differences were non-significant (p = 0.05). Very recently, two studies combining animal and human experiments suggested PEAR1 to be involved not only in platelet function, but also in platelet activation [15], megakaryopoiesis, and thrombopoiesis [24]. PEAR1 phosphorylation is a key event in a signaling cascade whereby platelet activation is amplified through sustained GP IIb/IIIa activation [15]. This hypothesis is in accordance with our finding of reduced platelet aggregation in carriers of the variant A allele. However, this seems to contrast the increased level of P-selectin seen in A allele carriers. Interestingly, the increased platelet activation level in A allele carriers may explain, at least in part, why these patients experienced a significantly higher number of cardiovascular events in the study by Lewis *et al* [13]. Coherence between increased levels of platelet aggregation and platelet activation seems plausible and has previously been demonstrated [25]. Therefore, the biological basis for the seemingly counter-intuitive findings reported herein is unknown and should be interpreted with caution. Firstly, the finding was not statistically significant (Table 4). Secondly, different SNPs in partial linkage disequilibrium with rs12041331, but in minimal linkage disequilibrium with each other may independently mediate the opposite effects. Thirdly, while rs12041331 seems to influence platelet aggregation levels, differences in P-selectin levels may instead result from rs12041331-mediated effects on vascular endothelial cells in which both PEAR1 and P-selectin are abundantly expressed.

We did not find an association between on-aspirin platelet aggregation and another *PEAR1* SNP (rs2768759), which contrasts a recent study showing increased platelet aggregation in C allele carriers [26]. Contrary to our study, this study population was of mixed ethnicity and included young healthy aspirin-treated siblings and offspring of probands with CAD. The study thus investigated an entirely different clinical setting. Similarly, we did not find consistent associations between platelet aggregation and any other SNP investigated, which is in perfect agreement with a recent comprehensive review in which *PEAR1* SNPs were not included [27]. After systematically reviewing a total of 50 SNPs, the authors concluded that only the Pl^A1/A2^ SNP (rs5918) might be associated with on-aspirin platelet aggregation in drug-naïve healthy individuals, but not in patients with cardiovascular disease [27]. Heterogeneity in terms of study cohorts, co-medication, and platelet function tests likely explains why few, if any, genes have been reproducibly associated with aspirin response before the discovery of rs12041331 in *PEAR1*. Furthermore, it remains to be clarified how aggregation-genotype associations are most effectively adapted into clinical practice [28]. In selected patients, current clinical guidelines advocate genotyping for genetic variants associated with clopidogrel response (class IIb recommendation), but in the context of aspirin the level of evidence does not yet justify clinical decision-making based on genotyping [29].

In general, the minor allele frequencies found in the present study were as expected [20], except that the rs12041331 A allele frequency was substantially lower in our study (8% versus 28%). While not matching the NCBI SNP database, in which allele frequencies are derived from the 1000 Genomes Project [30], our rs12041331 genotype distribution compares very well with that observed in recent comparable studies addressing the association between on-aspirin platelet aggregation and rs12041331 (minor allele frequencies ranging from 9% to 10%) [11]–[13]. This may partly reflect that these study populations consist of selected cardiovascular patients in whom the rs12041331 SNP has pronounced effect. Another explanation may be that individuals included in the 1000 Genomes Project are of mixed ethnicity, while our study cohort was Caucasian.

### Strengths and Limitations

Particular strengths of this exploratory study are the standardized inclusion of study participants, the unambiguous verification of aspirin adherence, and the use of two thoroughly evaluated platelet function tests.

A number of limitations deserve consideration when interpreting the results of the study. We analyzed our data according to the general genetic model, in which three distinct genotypes are retained [19]. This model, which makes no assumptions about how the risk for heterozygotes compares with that of homozygotes, requires an additional degree of freedom making it less statistically powerful than models in which heterozygotes are merged with either of the homozygote groups [19]–[31]. Moreover, to adjust for multiple comparisons and to reduce the type I error risk, we used a Bonferroni-adjusted significance level. This is a conservative approach, in particular when SNPs or traits are related [32]. Therefore, our results will tend to underestimate the true association between candidate gene variants and on-aspirin platelet aggregation. The sample size (n = 985) might not allow the identification of genetic variants that are only weakly associated with on-aspirin platelet aggregation. Moreover, the number of rs12041331 variant alleles was low, although a consistent association with platelet aggregation was observed for this SNP. No specific off-target control agonist (*e.g.* adenosine diphosphate or thrombin) was used to verify COX-1 independent effects of aspirin. We used citrated blood with the Multiplate Analyzer, although hirudin is recommended by the manufacturer. However, this is unlikely to have affected the observed aggregation-genotype associations, as results obtained with citrated and hirudinized blood correlate very well [33]. We did not assess off-aspirin platelet aggregation, as this was considered unethical in a cohort of patients with CAD. Thus, we do not know the patients' intrinsic platelet reactivity, which is known to be an important determinant of on-treatment platelet reactivity [34]. Finally, in the absence of data on clinical outcome, we could not determine if any of the investigated SNPs may confer an increased risk of adverse clinical events.

## Conclusion

In this study, a common genetic variant in *PEAR1* (rs12041331) influenced platelet aggregation in high-risk CAD patients treated with aspirin. The effect of this polymorphism seems non-COX-1 dependent, but it may be related to changes in platelet activation. The role for *PEAR1* genotyping in individualizing antiplatelet strategies merits further investigation. A total of 14 genetic variants previously suggested to influence aspirin efficacy were not associated with on-aspirin platelet aggregation.
